# Patient-Friendly Simplifications of Outpatient Reports Using Large Language Models via Few-Shot Prompting: Blinded Comparative Evaluation

**DOI:** 10.2196/101882

**Published:** 2026-11-06

**Authors:** Timo Elias Reimann, Vanessa Haug, Filippo Maria Verri, Christoph Leinert, Tim Fleiner, Michael Denkinger, Thomas Derya Kocar

**Affiliations:** 1 Institute for Geriatric Research Ulm, Ulm University Medical Center, Zollernring 26, Ulm, 89073, Germany, 49 7311870; 2 Department of Physiotherapy, Institute of Medical Engineering and Mechatronics, Ulm University of Applied Sciences, Ulm, Germany

**Keywords:** few-shot prompting, large language models, simplification, patient communication, medical report, outpatient clinic report, artificial intelligence, AI

## Abstract

**Background:**

Effective communication between clinicians and patients plays an important role in the successful delivery of health care. In geriatric medicine, where multimorbidity, polypharmacy, and functional limitations often make treatment plans complex, AI simplifications of clinical reports into patient-friendly language offer a promising way to improve communication.

**Objective:**

This study investigated whether AI could generate patient-friendly simplifications of outpatient reports that were comparable to those authored by human experts.

**Methods:**

Gemma-3-27B-it, an instruction fine-tuned open-source large language model (LLM) by Google, was used, and few-shot prompting was applied to enable the generation of patient-friendly simplifications of 64 deidentified geriatric outpatient reports written in German while preserving clinical meaning. LLM-generated simplifications were compared with those produced by 2 medical experts in a blinded evaluation. Quality was assessed using three approaches: (1) embedding-based semantic similarity to the original report, (2) blinded expert review by 2 clinicians, and (3) review by patient representatives, with the latter 2 using a standardized Likert-scale questionnaire.

**Results:**

AI-generated simplifications were comparable to geriatrician-authored simplifications regarding semantic similarity (*P*=.02) and were preferred by patient representatives (*P*<.001) as well as one expert reviewer (*P*=.001). Expert reviewers perceived physician-authored simplifications as more complete (*P*<.001), a finding that correlated with text length (*P*<.001).

**Conclusions:**

This study demonstrates the feasibility of using LLMs via few-shot prompting to simplify medical reports for improved perceived patient comprehension in geriatric care. Future prospective studies should examine whether such AI-driven simplifications positively impact objective health-related outcomes, adherence, and patient engagement.

## Introduction

Effective communication between clinicians and patients plays an important role in the delivery of health care [1], particularly in geriatric medicine, where adherence to complex medication and nonpharmacological recommendations directly influences the prevention of adverse events and the preservation of quality of life [2]. While medical reports containing diagnoses and recommendations are essential for communication between specialists and primary care physicians, their reliance on technical terminology can create a barrier to comprehension for older adults [3,4]. This gap between clinical language and patient health literacy can lead to uncertainty regarding health status and treatment plans [5], which in turn may affect patient compliance. Research indicates that poor health literacy is associated with reduced medication adherence, decreased engagement with lifestyle modifications, and lower rates of follow-through with specialist referrals [6]. For older adults facing multiple chronic conditions and complex medication regimens, this barrier can compromise treatment effectiveness [7] and increase the risk of adverse events.

Current literature has explored the simplification of radiology and pathology reports into accessible language [8,9], suggesting that AI-based systems can perform these tasks. However, a gap remains regarding the adaptation of physician correspondence such as outpatient reports or discharge letters, which often combine diagnoses, multimorbidity, medication changes, functional assessments, and follow-up recommendation components central to long-term treatment adherence and continuity of care. Furthermore, evaluation methodologies in existing studies have relied predominantly on assessments by specialist physicians rather than direct patient feedback [10].

Addressing this gap provides an opportunity to improve patient outcomes in geriatric care. Large language models (LLMs) and few-shot prompting may enable the simplification of complex medical reports into patient-friendly language while preserving clinical accuracy. Few-shot prompting is a technique in which a limited set of illustrative examples is provided within the input prompt to guide an LLM toward the desired output format and task performance [11]. Therefore, the objective of this study was to evaluate whether an open-weight LLM using a structured few-shot prompting configuration could effectively simplify complex geriatric medical reports while maintaining clinical accuracy and achieving a quality level comparable to that of medical professionals.

## Methods

### Study Setting and Data Source

This study was conducted at the Geriatrische Instituts- und Sturzambulanz (GISA), a specialized outpatient clinic at the Agaplesion Bethesda Clinic Ulm, Germany. The GISA provides comprehensive fall risk assessment and management for older adults according to current prevention guidelines. The patient population consists of adults aged 65 years and older presenting with at least one geriatric syndrome. These patients are characterized by frailty and multimorbidity, most commonly presenting with multifactorial gait disorders. Deidentified medical reports from 64 patients who were treated at the clinic between January 1, 2023, and August 27, 2025, were obtained. Outpatient reports are defined as clinical reports of a single outpatient visit. Each report typically includes the main and other diagnoses, patient anamnesis, clinical findings, specific diagnostic procedures (including instrumental diagnostics), and laboratory findings, as well as a clinical summary encompassing the clinical reasoning and a structured list of recommendations and follow-up procedures. These reports, written in German, represented a diverse range of geriatric patient cases presenting with fall-related concerns and served as the data source for the simplification process. To prepare the input for the model, the sections for main diagnoses, other diagnoses, clinical summary, and procedures were extracted from each outpatient report and concatenated into a single continuous string. No missing data were identified across the extracted parameters; the documentation of these elements is a mandatory component of the outpatient clinical reporting process.

### Study Design and Reporting

The present study was a single-center, retrospective, blinded comparative evaluation of an LLM-based document simplification tool for geriatric outpatient reports. This study is reported with reference to the TRIPOD+LLM (Transparent Reporting of a Multivariable Prediction Model for Individual Prognosis or Diagnosis–Large Language Model) framework for health care LLM studies [12]; the completed checklist is provided in Checklist 1. As the study did not evaluate a diagnostic or prognostic prediction model, checklist items related to prediction probabilities, outcome events, and model updating were considered not applicable.

### AI Simplification Tool and Development

The simplification tool was developed using an LLM integrated via a WebUI interface (Hassan Draga; text-generation-webui). During a piloting phase, several open-source models, including GLM-4.5-Air (Z.ai) [13], Qwen3-32B (Alibaba Cloud) [14], NVIDIA-Nemotron-Nano-9B-v2 [15], and MedGemma-27B-text-it (Google DeepMind) [16], were evaluated for their instruction-following capability and response quality. To ensure consistency, all candidate models were tested using the same prompt, which was iteratively refined. On the basis of comparative testing, Gemma-3-27B-it by Google [17] was selected as the final model; the primary criterion was its ability to consistently follow complex instructions, with additional criteria including factual correctness; eloquence; and a balanced, patient-appropriate tone. This development process was led by the first author (TER), who iteratively refined the prompt using optimization suggestions from the LLMs and feedback from 2 working group meetings regarding the preferred output template. The simplifications targeted the health literacy of older adults without a professional health care background to ensure accessibility for the general geriatric population. The final implementation used a structured few-shot prompting approach [10]. The corresponding prompt was developed through an iterative process incorporating human-written simplifications and optimization suggestions provided by the LLMs. The final prompt consisted of general behavioral guidelines, 4 input-output examples to demonstrate the desired style and formatting, and a predefined blank template to ensure consistency (Multimedia Appendix 1). The 4 few-shot examples were written by the first author (TER), were entirely distinct from the 64 reports used in the evaluation set, and were not produced by the simplifiers or raters. The final prompt was fixed across all inferences. To optimize model performance, XML-style markup was used to segment instructions, and the structural template was positioned at the end of the prompt to leverage the recency effect of the LLM [18,19]. The output produced by the model was the plain-language version of the document, strictly adhering to the format specified by the predefined template. The model was deployed locally on an NVIDIA RTX A6000 (Ampere generation) graphics processing unit (GPU) using 8-bit quantization via the ggml framework (q8_k_xl format).

### Simplification and Evaluation Procedure

A dual-simplification protocol was used to establish a comparative baseline. All 64 reports were simplified by the developed AI tool. Physician-authored simplifications were produced by 2 specialist physicians, each simplifying 32 nonoverlapping reports adhering to the predefined blank template. To prevent bias, a strict separation of concerns was maintained: model development (TER, doctoral student, in training), the creation of expert simplifications (FMV, internist and geriatrician in training, 8 years of professional experience; TDK, neurologist and geriatrician, 13 years of professional experience), and the rating process (VH, sports scientist in geriatric health care, 4 years of professional experience; CL, neurologist and geriatrician, 15 years of professional experience) were kept independent, with no information shared between these groups except for the predefined blank template. Specifically, the expert simplifiers had no insight into the few-shot prompt, nor were they given constraints regarding text length or structure.

The semantic similarity of the simplifications relative to the original medical reports was measured using an embedding-based analysis with the Qwen3-Embedding-8B model [20], quantified as cosine similarity to the original document. An embedding model maps text into vectors within a vector space, where semantically similar vectors share a common direction. This relationship can be quantified using the cosine similarity between 2 vectors [21]. This metric serves as a proxy for semantic similarity in embedding space rather than a direct measure of factual correctness or simplification fidelity. Following paraphrase detection benchmarks, and as a pragmatic proxy for semantic fidelity, a cosine similarity of 0.7 or higher was considered indicative of semantic similarity [20], a threshold that is also supported by empirical analyses of transformer-based embedding models for paraphrase detection [22]. To determine whether the AI-generated simplifications were comparable to the human simplifications, a paired *t* test of equivalence was performed [23]. The equivalence margin was defined as the mean absolute difference in cosine similarity between the 2 independent human simplifiers. In other words, AI simplifications were considered comparable if their semantic similarity to the original text did not differ from that of human simplifications by more than the difference observed between human simplifiers.

The simplifications were reviewed by independent medical experts and patient representatives, each in a randomized order. The patient representatives (n=64; age range 65-91 years; women-to-men ratio of approximately 2:1) were residents of the Ulm area and were recruited from 3 settings: members of the *Generationentreff* (intergenerational meeting center), residents of assisted living facilities, or patients treated at the fall clinic of the Agaplesion Bethesda Clinic during the study period. Each patient representative performed a single rating. None of the patient representatives possessed any prior medical training or professional health care experience. Both groups used standardized questionnaires featuring Likert scales adapted from the instrument used by Stephan and colleagues [24]. The original patient questionnaire included the items “clarity,” “tone,” “structure,” and “patient engagement.” For the current study, “clarity” and “tone” (rephrased as “appropriateness of the language”) were retained. The items “patient engagement” and “structure” were omitted as the former was deemed not measurable in this context and the latter was predefined. To further refine the evaluation, we introduced “appropriateness of content” and “positivity of content” for patient representatives as well as “completeness” and “correctness” for expert reviewers. In both questionnaires, the “appropriateness of the language” served as the operational measure for whether the text was truly patient-friendly for an older adult without a medical background. Interrater reliability was assessed using the intraclass correlation coefficient, and results are reported in Multimedia Appendix 1.

### Statistical Analysis

For the statistical analysis of these questionnaires, individual Likert-scale items were aggregated (range 4-20 points) and compared using 2-tailed paired *t* tests, while an exploratory analysis of individual items was conducted using Wilcoxon signed rank tests. As part of an error analysis, further multivariable regression analyses were used to evaluate potential predictors and their interactions, when results warranted further investigation. Specifically, for the overall score, an ordinary least squares (OLS) regression was used, and for Likert-scale results, an ordinal regression was used. Because each source report contributed 1 AI-generated and 1 human-authored simplification, the paired data structure was accounted for at the report level. As an additional analysis, a per-simplifier comparison was conducted; results are provided in Multimedia Appendix 1.

All statistical analyses were performed using Python (version 3.12.2; Python Software Foundation), specifically the SciPy (version 1.12.0) and statsmodels (version 0.14.4) libraries. Effect sizes are reported as Cohen *d*, rank-biserial *r*, ρ, or odds ratio (OR) depending on the test. In instances where 95% CIs were not directly provided by the model, they were estimated via bootstrapping with 1000 iterations. Statistical significance was defined as *P*<.05. The complete analysis code is made available on GitHub [25].

### Ethical Considerations

This study was approved by the Ethics Committee of Ulm University (reference number 150/25-MO/bal.) and conducted in accordance with the principles of the World Medical Association Declaration of Helsinki [26]. All patients provided written informed consent before participation. All study data were deidentified to protect patients’ privacy and confidentiality. Participants received no compensation for their involvement in the study.

## Results

The model strictly adhered to the required formatting in all 64 cases without need for manual modification.

### Semantic Similarity

To evaluate the semantic similarity of the simplifications relative to the original medical reports, cosine similarity was measured using an embedding-based analysis and used as a proxy. Both groups exceeded the quality threshold of 0.7, with mean cosine similarity scores of 0.767 (SD 0.041) for human simplifications and 0.749 (SD 0.041) for AI-generated simplifications. To determine whether the AI-generated simplifications were comparable to the human simplifications, a paired *t* test of equivalence was performed [23], with the equivalence margin defined by the mean absolute difference observed between 2 independent human simplifiers. This analysis demonstrated that the AI simplifications were comparable to human simplifications regarding semantic similarity to the original document (*P*=.02; Cohen *d*=0.379, 95% CI 0.163-0.605; interhuman Cohen *d*=0.482, 95% CI 0.153-0.911). A further per-simplifier analysis is provided in Tables S2 and S3 in Multimedia Appendix 1, confirming that the AI’s semantic similarity of the simplifications relative to the original medical report was comparable to the variability observed between human experts.

### Overall Quality Assessment

The overall quality of the simplifications was assessed by patient representatives and 2 independent medical experts (Table 1 and Figure 1). Patient representatives rated AI-generated simplifications significantly higher than human simplifications (*P*<.001). Among the expert reviewers, expert 1 (sports scientist) rated AI simplifications significantly higher (*P*=.001), whereas no significant difference was observed in the ratings by expert 2 (geriatrician; *P*=.15; Table 1).

**Table 1.** Overall quality assessment of human-authored vs AI-generated simplifications^a^.

Raters	Human-authored simplifications, mean (SD)	AI-generated simplifications, mean (SD)	Cohen *d* (95% CI)	*P* value
Patient representatives	14.953 (4.274)	17.266 (3.123)	–0.478 (–0.739 to –0.243)	<.001
Expert 1 (sports scientist)	16.766 (1.925)	17.891 (1.416)	–0.448 (–0.705 to –0.216)	.001
Expert 2 (geriatrician)	14.516 (2.475)	13.922 (2.339)	0.185 (–0.063 to 0.473)	.15

a The possible score range was 4 to 20 points. Mean scores and SDs are provided as evaluated by the patient group and 2 independent expert raters, including the corresponding *P* values for statistical significance, as well as the Cohen *d* and its 95% CI.

**Figure 1.**
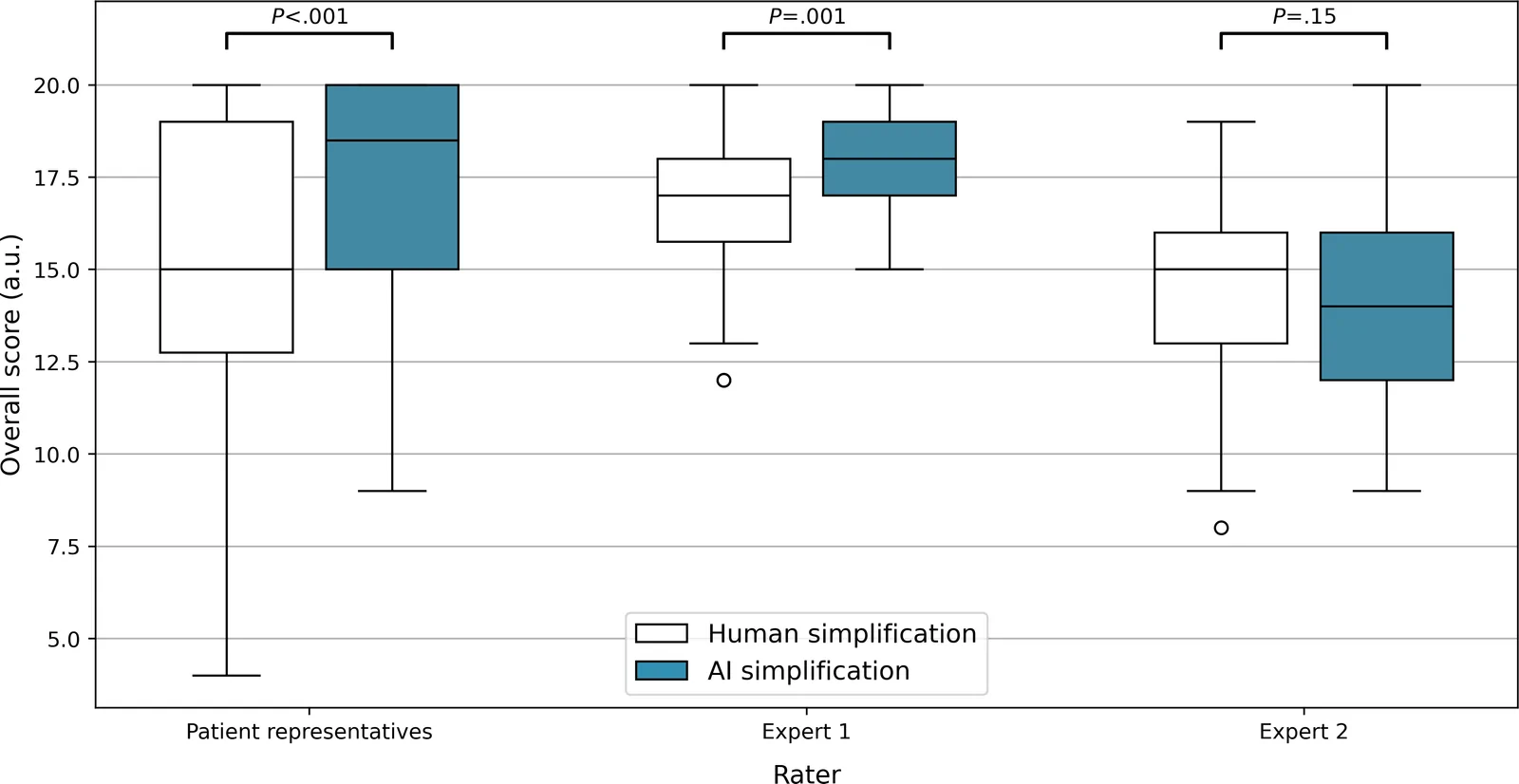
Overall quality scores for human and AI simplifications across rater groups. Individual Likert-scale items were aggregated to an overall score (range 4-20 points). The patient representative mean score was 14.953 (SD 4.274) for human-authored simplifications and 17.266 (SD 3.123) for AI-generated simplifications. Mean scores from the first expert rater were 16.766 (SD 1.925; human authored) and 17.891 (SD 1.416; AI generated). Mean scores from the second expert rater were 14.516 (SD 2.475; human authored) and 13.922 (SD 2.339; AI generated). Outliers were observed in both expert ratings but exclusively within the human-authored simplifications. a. u.: arbitrary units.

### Exploratory Analysis of Individual Items

An exploratory analysis of individual Likert-scale items revealed specific differences in perception between the raters (Table 2). Patient representatives rated AI simplifications significantly higher across all assessed dimensions, including clarity, appropriateness of content, positivity of content, and appropriateness of language (*P*≤.01 in all cases). Among the expert reviewers, results were divergent: expert 1 (sports scientist) rated AI simplifications significantly higher in terms of clarity (*P*<.001), correctness (*P*=.01), and appropriateness of language (*P*<.001) but rated human simplifications as significantly more complete (*P*=.001). Expert 2 (geriatrician) found no significant difference in clarity (*P*=.06), correctness (*P*=.21), or appropriateness of language (*P*=.08) but similarly rated human simplifications as significantly more complete (*P*<.001). Table 2 provides effect sizes and CIs. A positive correlation was observed between the perceived completeness and overall text length (expert 1: Spearman ρ=0.291, 95% CI 0.119-0.439, and *P*=.001; expert 2: Spearman ρ=0.246, 95% CI 0.078-0.396, and *P*=.005). Human simplifications were longer than AI-generated texts (2510, SD 1079 vs 1523, SD 163 characters; Cohen *d*=0.933, 95% CI 0.752-1.145; *P*<.001). However, ordinal regression analysis showed that neither authorship type (AI vs human; expert 1: OR 0.166, 95% CI 0.002-18.496, and *P*=.46; expert 2: OR 0.775, 95% CI 0.009-64.718, and *P*=.91) nor its interaction with length (per 100 characters; expert 1: OR 1.060, 95% CI 0.787-1.429, and *P*=.70; expert 2: OR 0.945, 95% CI 0.712-1.256, and *P*=.70) significantly predicted completeness. OLS regression analysis indicated that authorship type (AI vs human; β=5.178, 95% CI –3.903 to 14.260; *P*=.26), text length (per 100 characters; β=–0.041, 95% CI –0.127 to 0.046; *P*=.35), or their interaction (β=–0.215, 95% CI –0.794 to 0.364; *P*=.47) did not significantly predict the patient representatives’ overall score.

**Table 2.** Exploratory analysis of individual items^a^.

Items	Human-authored simplifications (4-20), median (IQR)	AI-generated simplifications (4-20), median (IQR)	Rank-biserial, *r* (95% CI)	*P* value
Patient representatives				
Clarity	4 (3.0 to 5.0)	5 (4.0 to 5.0)	–0.642 (–0.891 to –0.352)	.001
Appropriateness of the content	4 (3.0 to 5.0)	5 (4.0 to 5.0)	–0.663 (–0.880 to –0.389)	.001
Positivity of the content	4 (3.0 to 5.0)	5 (4.0 to 5.0)	–0.474 (–0.760 to –0.157)	.01
Appropriateness of the language	4 (3.0 to 5.0)	5 (4.0 to 5.0)	–0.582 (–0.820 to –0.271)	.001
Expert 1 (sports scientist)				
Clarity	4 (4.0 to 5.0)	5 (5.0 to 5.0)	–0.899 (–1.000 to –0.741)	<.001
Completeness	5 (4.0 to 5.0)	4 (4.0 to 5.0)	0.527 (0.248 to 0.777)	.001
Correctness	4 (4.0 to 4.0)	4 (4.0 to 5.0)	–0.440 (–0.737 to –0.131)	.01
Appropriateness of the language	4 (4.0 to 5.0)	5 (4.0 to 5.0)	–0.821 (–0.946 to –0.636)	<.001
Expert 2 (geriatrician)				
Clarity	4 (3.0 to 4.0)	4 (3.75 to 4.0)	–0.333 (–0.669 to 0.014)	.06
Completeness	4 (3.0 to 4.0)	3 (2.0 to 4.0)	0.712 (0.488 to 0.890)	<.001
Correctness	4 (3.0 to 4.0)	3 (3.0 to 4.0)	0.215 (–0.137 to 0.553)	.21
Appropriateness of the language	4 (3.0 to 4.0)	4 (4.0 to 4.0)	–0.326 (–0.655 to 0.042)	.08

a Median scores and IQRs are provided for specific assessment criteria, including clarity, appropriateness, positivity, completeness, and correctness, comparing human-authored and AI-generated simplifications as evaluated by the patient group and 2 expert raters, with accompanying *P* values for statistical significance, as well as the rank-biserial correlation coefficient (*r*) and its corresponding CI.

## Discussion

### Principal Findings

The primary finding of this study is that AI-generated simplifications, powered by Gemma 3-27B-it and few-shot prompting, were comparable to professional human simplifications and preferred by patient representatives across all evaluation metrics. The AI simplifications were comparable in semantic similarity relative to the original medical documents while being preferred by patient representatives and one of the two expert raters. These results suggest that LLMs can preserve the core content of complex geriatric reports while delivering them in a format that is perceived as more accessible to the patient.

The preference observed among patient representatives highlights a gap in traditional physician-authored simplifications. While clinicians are trained to prioritize exhaustive clinical completeness, they often use a professional vocabulary, raising uncertainty in lay audiences [5]. In contrast, the AI’s ability to synthesize information into a concise, jargon-free format is rated more favorably by older adults. This supports the notion that in patient communication, brevity can enhance comprehension provided the core clinical meaning remains intact. However, the divergent views among expert reviewers regarding completeness provide a more nuanced perspective. Both experts rated human simplifications as more complete, pointing to a tendency of the AI to omit clinical details. This difference in perceived completeness corresponds to a significant difference in text length as human simplifications were substantially more voluminous, and overall text length positively correlated with completeness scores. Conversely, the brevity of the AI simplification may in part be due to conciseness constraints within the prompt. When modeling the interaction between source and length, ordinal regression did not show evidence that the association between length and completeness differed significantly between AI and human simplifications. Similarly, OLS regression indicated that authorship type, text length, or their interaction did not significantly influence the preference scores provided by patient representatives. Taken together, these findings suggest that the perceived differences in completeness are a conciseness-completeness trade-off, with neither humans nor AI having an advantage. To evaluate the clinical impact of these omissions, the study group assessed whether any crucial information, defined as data having an immediate and direct impact on patient treatment, had been lost. This review was conducted by two raters (VH and TDK), with any disparities resolved through a joint conference including a third rater (TER). Of the 48 cases rated as inferior in completeness by at least one reviewer, crucial information was omitted in only 3 instances (1 magnetic resonance imaging recommendation and 2 inpatient referrals). Notably, these 3 specific omissions were no longer observed following an exploratory update to the newer Gemma 4 model by Google released after the study’s completion. While this informal post hoc observation provides an example indication of the model’s capacity for improvement, a more rigorous evaluation of clinical safety is warranted in future research. Other identified omissions, such as screening protocols, follow-up examinations, or differential diagnostics for rare diseases, were common but not considered crucial. Instead, the AI tended to prioritize actionable advice for the patient. While this brevity may reduce the patient’s cognitive load, the clinician may perceive it as an information loss. This balance could potentially be adjusted by refining the few-shot prompt to emphasize specific clinical categories that must not be omitted, such as referrals, diagnostic follow-up, medication changes, and safety-relevant recommendations. These findings underscore that human oversight remains necessary to ensure clinical safety. Future work should incorporate structured key clinical fact retention or human fidelity assessments, particularly for referrals, diagnostic follow-up, medication changes, and safety-relevant recommendations.

The present study aligns with and extends recent research on AI-supported simplification of medical reports. AI-interpreted spine magnetic resonance imaging reports were shown to improve patient comprehension, clarity, and satisfaction compared to standard reports in a prospective single-center study, although some omissions or inaccuracies occurred [9]. The present study differs in two key aspects: first, we compared AI-generated simplifications against human-authored simplifications, providing a more rigorous benchmark. Second, the present study’s focus was geriatric outpatient medicine, involving complex reports on multimorbidity, functional status, and polypharmacy rather than a single imaging modality. The capacity of AI to handle complex clinical data is further evidenced by the fact that iterative simplification of dental radiology reports significantly improves readability and patient-rated clarity [24]. The potential for increased clinical efficiency is further highlighted by Yang and colleagues [10], who found that GPT-4–generated pathology reports significantly improved patient understanding while reducing physician-patient communication time from 35 to 10 minutes. In the context of geriatrics, where the intricacy of multimorbidity often makes consultations exceptionally time-consuming, such a reduction in basic terminology explanation could allow clinicians to focus more on targeted counseling and shared decision-making. By extending this application to geriatric care and indicating a similar preference among patient representatives, the present study reinforces the versatility of LLMs in bridging communication gaps across diverse medical disciplines. While most LLM evaluations are based on English-language datasets, the present study demonstrates that few-shot prompting effectively simplifies German-language geriatric reports. This suggests feasibility in this specific setting, although broader linguistic and clinical generalizability remains to be tested. Together, these findings demonstrate that AI-generated simplifications are semantically comparable to human-authored versions and are preferred by patient representatives, supporting their further integration into clinical workflows.

A key technical contribution of this work is the demonstration that few-shot prompting is an effective method for patient-oriented simplifications of outpatient reports. This approach allows for the deployment of simplification tools without the need for expensive fine-tuning; while the prompt requires an initial iterative optimization, this represents a one-time development investment that ensures consistent performance across all reports. By benchmarking this automation against actual clinical workloads rather than an idealized human benchmark, we demonstrate a pragmatic reduction in the recurring operational burden of medical documentation. In this study, manual simplification took approximately 30 minutes per report compared to approximately 3 minutes for automated generation, although these durations were not formally measured. Furthermore, the use of Gemma-3-27B-it, a model with 27 billion parameters, is noteworthy because it can be executed on consumer-grade hardware such as the NVIDIA RTX A6000 (Ampere generation) GPU used in the present study. This enables clinics to run the simplification process locally and entirely offline, addressing data privacy concerns. Overall, this study serves as a proof of concept demonstrating that AI-generated simplifications already yield promising results. Given that the effectiveness of few-shot prompting benefits from the general advancement of LLM technology, these outcomes may be further optimized through the continued refinement of both the prompting strategy and the underlying models. Furthermore, while a standardized prompt was used here to maintain a controlled design, LLMs enable patient-specific individualization. Tailoring language complexity to an individual’s reading level, educational level, or primary language represents a promising direction for future research.

Significant differences were observed between the human simplifiers regarding both patient representative ratings (Table S4 in Multimedia Appendix 1) and semantic similarity (Multimedia Appendix 1). These findings highlight the inherent variability in expert simplification. The variance in semantic similarity underscores that the difference between human experts exceeded the difference between human and AI performance.

### Limitations

Several limitations must be acknowledged. First, this study was conducted within a single specialized geriatric fall clinic, which may limit the generalizability of the findings. Second, the evaluation relied on a sample of 64 reports, and the human comparator was produced by two physicians who each simplified 32 different reports. Because these report sets were nonoverlapping, potential differences in case mix, report complexity, and individual writing styles may have influenced the human comparator. Consequently, intersimplifier variability could not be estimated, and the pooled human condition may partly reflect simplifier-specific style, detail orientation, and wording preferences. Third, the observed variability between human simplifiers indicates that interexpert differences may influence the comparative results. Fourth, human simplifiers were not provided with the few-shot prompt and received no specific text length limitations. This led to significant differences in text length, which may have influenced the ratings. Fifth, the omission of information with immediate treatment relevance in some AI-generated simplifications highlights the ongoing requirement for human verification to ensure patient safety. Sixth, preference scores may be influenced by the heterogeneity of health literacy among older adults (see the variance in patient ratings in Tables 1 and 2), and this study did not assess objective comprehension, recall, anxiety, adherence, or clinical outcomes. Seventh, the present study reflects the preferences of patient representatives rather than the original patients. While both groups share similar demographic and clinical profiles, individual health literacy and the context of receiving one’s own report may influence the perception of clarity and acceptability. Eighth, semantic similarity provides a scalable proxy for fidelity but cannot replace a rigorous clinical safety review. The occurrence of clinically important omissions demonstrates that high semantic similarity does not automatically guarantee clinical completeness or safety. Finally, the long-term impact of these simplifications on patient health outcomes was not measured; however, an intervention trial is currently planned with patient adherence as the primary end point alongside several secondary health-related outcomes.

### Conclusions

This study demonstrates that AI-generated simplifications are preferred by patients over human-authored simplifications while maintaining comparable semantic similarity relative to the original medical report. Furthermore, the expert evaluations showed a tendency toward the AI-generated simplifications and identified a trade-off between conciseness and completeness. These results demonstrate the feasibility of using few-shot prompting with open-weight LLMs as a scalable approach to support patient communication in geriatric care, providing a foundation for further prospective evaluations.

## Supplementary material

10.2196/101882 Multimedia Appendix 1 Supplementary materials including templates, evaluation questionnaires, fictional example, few-shot prompt, and supplementary analyses, figures, and tables.

10.2196/101882 Checklist 1 TRIPOD+LLM checklist.

## Abbreviations

GISA: Geriatrische Instituts- und Sturzambulanz
GPU: graphics processing unit
LLM: large language model
OLS: ordinary least squares
OR: odds ratio
TRIPOD+LLM: Transparent Reporting of a Multivariable Prediction Model for Individual Prognosis or Diagnosis–Large Language Model
