# Supplementary material for: Patient-Friendly Simplifications of Outpatient Reports Using Large Language Models via Few-Shot Prompting: Blinded Comparative Evaluation
Source: J Med Internet Res. 2026 Nov 6;28:e101882. doi: 10.2196/101882 (PMC13742778; doi:10.2196/101882)
Supplement: Supplementary file 1 [file jmir-v28-e101882-s001.docx]

**Patient-Friendly Simplifications of Outpatient Reports Using Large Language Models via Few-Shot Prompting: Blinded Comparative Evaluation**

Multimedia Appendix 1

# Blank template

The following is the blank template utilized in the study. For the benefit of international readers, we provide the original German version alongside its English translation.

## German

Sehr geehrte ,

hier ist eine KI-generierte Übersetzung Ihres Arztbriefes aus der Sturzambulanz. Das Ziel ist es, Ihnen die wichtigsten Punkte in klaren, einfachen Worten zu erklären, damit Sie sich gut informiert und sicher fühlen.

**Zusammenfassung Ihrer wichtigsten Diagnosen:**

| **Diagnose** | **Erklärung** |
| --- | --- |
|  |  |
|  |  |
|  |  |
|  |  |
|  |  |

**Weitere bekannte Erkrankungen:**


**Was wurde bisher gemacht?**

**Was sollten Sie jetzt tun?**

| **Maßnahme** | **Erklärung** | **Checkliste** |
| --- | --- | --- |
|  |  |  |
|  |  |  |
|  |  |  |
|  |  |  |
|  |  |  |
|  |  |  |

**Wichtige Kontaktdaten:**
Geriatrische Instituts- und Sturzambulanz:
📞 0731 187-181
📧 [sturzambulanz.bgu@agaplesion.de](mailto:sturzambulanz.bgu@agaplesion.de)
🌐 [www.sturzimpfung-ulm.de](https://www.sturzimpfung-ulm.de)

Ich hoffe, diese Übersetzung hilft Ihnen weiter. Bei Fragen oder Unsicherheit sprechen Sie bitte mit Ihrem Arzt.

Mit freundlichen Grüßen,

GISelA 🌼 (Ihre KI-Assistentin)

## English

Here is an AI-generated translation of your medical report from the Fall Clinic. The goal is to explain the most important points to you in clear, simple words, so that you feel well-informed and secure.

Summary of your most important diagnoses:

| **Diagnosis** | **Explanation** |
| --- | --- |
|  |  |
|  |  |
|  |  |
|  |  |
|  |  |

**Other known conditions:**

**What has been done so far?**

**What should you do now?**

| **Measure** | **Explanation** | **Checklist** |
| --- | --- | --- |
|  |  |  |
|  |  |  |
|  |  |  |
|  |  |  |
|  |  |  |
|  |  |  |

Important contact details:
Geriatric Institute and Fall Clinic:
📞 0731 187-181
📧 sturzambulanz.bgu@agaplesion.de
🌐 www.sturzimpfung-ulm.de
Memory Clinic ZfP Südwürttemberg: 📞 0731 187 5317

I hope this translation is helpful to you. If you have any questions or uncertainties, please speak with your doctor.

Sincerely,

GISelA 🌼 (Your AI Assistant)

# Expert questionnaire

Presented below is the Expert Questionnaire in the original German version used in this study, accompanied by a translation for the international readership.

## German

**Bewertungsbogen für Experten**

**ID#**_____

|  | Stimme gar nicht zu (1) | (2) | (3) | (4) | Stimme voll und ganz zu (5) |
| --- | --- | --- | --- | --- | --- |
| Die Übersetzung ist verständlich. |  |  |  |  |  |
| Die Übersetzung ist vollständig. |  |  |  |  |  |
| Die Übersetzung ist korrekt. |  |  |  |  |  |
| Die Sprache der Übersetzung ist angemessen. |  |  |  |  |  |

Dankeschön für Deine Mühen :-)

## English

**Evaluation Form for Experts**

**ID#**_____

|  | Strongly disagree (1) | (2) | (3) | (4) | Strongly agree (5) |
| --- | --- | --- | --- | --- | --- |
| The translation is understandable. |  |  |  |  |  |
| The translation is complete. |  |  |  |  |  |
| The translation is correct. |  |  |  |  |  |
| The language used in the translation is appropriate. |  |  |  |  |  |

Thank you for your efforts :-)

# Patient questionnaire

Presented below is the Patient Questionnaire in the original German version used in this study, accompanied by a translation for the international readership.

## German

**Bewertungsbogen für Interessenvertretung**

**ID#**_____

|  | Stimme gar nicht zu (1) | (2) | (3) | (4) | Stimme voll und ganz zu (5) |
| --- | --- | --- | --- | --- | --- |
| Die Übersetzung ist verständlich. |  |  |  |  |  |
| Der Informationsgehalt der Übersetzung ist angemessen. |  |  |  |  |  |
| Ich nehme die Übersetzung als positiv und motivierend wahr. |  |  |  |  |  |
| Die Sprache der Übersetzung ist angemessen. |  |  |  |  |  |

Vielen Dank für Ihre Mitarbeit!

## English

**Evaluation Form for Patient Representative**

**ID#**_____

|  | Strongly disagree (1) | (2) | (3) | (4) | Strongly agree (5) |
| --- | --- | --- | --- | --- | --- |
| The translation is understandable. |  |  |  |  |  |
| The information content of the translation is appropriate. |  |  |  |  |  |
| I perceive the translation as positive and motivating. |  |  |  |  |  |
| The language used in the translation is appropriate. |  |  |  |  |  |

Thank you for your participation!

# Sham Example

### The following case describes a fictional patient. For the benefit of international readers, we have provided the original German entry as it would appear in the application, along with English translations of both the input and the corresponding output.

## German

### Input

Arztbrief von Herrn Maier:

Multifaktorielle Gangstörung

mit 1) sensorisch-ataktische Gangstörung

bei 1A) Polyneuropathisches Syndrom unklarer Genese DD malnutritiv DD idiopathisch

Empfehlung: weiterführende laborchemische und elektrophysiologische Abklärung ggf. Therapie

1B) Sekundär eingeblutete cerebrale Ischämie parietal und temporal rechts 07/22

mit residueller Beinataxie links, V.a. Störung der Raumwahrnehmung DD visueller Neglect

Empfehlung: Ergotherapie Einzel zum Training der Raumwahrnemung und Erlernen Kompensationsstrategien

1C) Z.n. Netzhautablösung RA (deutliche Defizite) und LA (geringe Defizite)

2) V.a. leichte apraktische Gangstörun

3) V.a. Mild cognitive impairment

Auffälligkeiten im Dual-Task Gang, im kognitiven Screening keine Auffälligkeiten

Empfehlung: Verlaufskontrolle mittels MoCA Test in 3-6 Monaten

4) Rezidivierende Stürze

zuletzt am 16. Juni 2024, mit Fraktur am Os triquetrum links.

5) Intermittierender AV-Block III°

Implantation eines Herzschrittmachers 06/2023

6) Paroxysmales Vorhofflimmern

Antikoagulation mit Apixaban

7) V.a. Osteoporose

8) Schwergradige obstruktive Schlafapnoe

9) Arterielle Hypertonie

10) Adipositas

11) Diabetes mellitus Typ 2, insulinpflichtig

12) V.a. Presbyakusis

Hörgeräteversorgung empfohlen

Im Rahmen der Vorstellung in der Geriatrischen Instituts- und Sturzambulanz fielen mehrere Komponente einer Gangstörung auf:

ad 1A)

Es fiel eine sensorisch-ataktische Gangstörung auf die a.e. multifaktoriell bedingt erscheint. Es fiel ein polyneuropathisches Syndrom aktuell unklarer Genese auf.

Wir empfehlen zur weiteren Abklärung eine laborchemische Diagnostik insb. bzgl. potenziell modifizierbarer Ursachen: Vitamin B12, Holotranscobalamin, Methylmalonsäure, Vit. B6, Vit B1, Folsäure, Serum-Ephorese (MGUS?), Vasculitisscreening (BSG, CRP; ANA, ANCA, Hepatitis C). Ein HbA1c Wert war in einer Laborentnahme aus 10/23 leicht erhöht bei 6,4%, der Pat. berichtet über eine Gewichtszunahme und vermehrtes Essen sei Beendigung des regelmäßige Alkoholkonsums, diesbzgl. sollte eine genauere Testung bzgl. eines Diabetes erwogen werden.

Wir empfehlen eine elektrophysiologische Untersuchung beim niedergelassenen Neurologen zur Sicherung der klinischen Diagnose.

Wir empfehlen zur Behandlung der sensorisch-ataktischen Gangkomponente: ein strukturiertes Sturzpräventionsprogramm z.B. Gruppenbasiert nach dem Ulmer Modell (2-3x/Wo 30-60min) mit gezielten Übungen für Gleichgewicht und Kraft mit regelmäßiger Anpassung des Schwierigkeitsgrads an das individuelle Niveau. Alternativ kann der Pat. auch ein Eigentraining in seinen Alltag integrieren, z.B. anhand des LiFE Trainings. (www.life-alltagsuebungen.de).

Zusätzlich ist ein Perturbationstraining z.B. auf einem Laufband zum Training von Ausfall uns Abfangschritten sinnvoll. Dieses kann im Rahmen der sog. SturzImpfung in unserer Ambulanz erfolgen und wurde von dem Pat. bereits zweimal genutzt mit subjektiver Besserung der Gangsicherheit. Bei Interesse und Fragen gerne Kontaktaufnahme über unsere Sturzambulanz unter Tel. 0731 187 181 oder Homepage www.sturzimpfung-ulm.de.

ad 1B) und 1C)

Es fielen klinisch Defizite im Rahmen der Raumwahrnehmung sowie eine Beinataxie links auf. Diese sind zum Einen auf die stattgehabte hochparietal gelegene cerebrale Ischämie und zum Anderen auf die Visustörung mit auch H.a. fingerperimetrischen Gesichtsfelddefekten zurückzuführen.

Wir empfehlen darüber hinaus ergänzend zu den unter ad 1) genannten Therapiemaßnahmen eine ergotherapeutische Einzeltherapie mit dem Ziel der Verbesserung der Raumwahrnehmung sowie ggf. Erlernen von Kompesnationsstrategien.

Wir empfehlen bzgl. der Visusstörungen auf das Tragen einer Gleitsichtbrille, die den Wechsel zwischen Nah- und Fernsicht ebenfalls erschweren kann, zu verzichten und stattdessen lieber eine einzelene Brille für Fern- und eine andere für Nahsicht zu verwenden.

ad 2)

Es fiel eine leichte apraktische Gangkomponente mit Verschlechterung der Parameter der Ganganalyse unter semantischen Doppel-Tätigkeits-Aufgaben (Dual task), jedoch noch unterhalb des Cut offs von 20%, auf. Diese können durch subklinische kognitive Defizite bedingt sein. Subjektiv berichtet der Pat. von langsam progredienten Hirnleistungsstörungen, die teilweise eine Relevanz für seinen Alltag haben. Ein kognitives Screening ergab hier keine messbaren Hinweise auf kognitive Defizite.

Wir empfehlen zur weiteren Abklärung eine Verlaufskontrolle mit einem für das hohe kognitive Ausgangsneveau des Patienten geeigneten kognitiven Kurztest (z.B. normaler MOCA Test) in 3-6 Monaten sowie die weitere Verlaufsbeobachtung bei anhaltenden oder progredienten Beschwerden ggf. Vorstellung in einer Gedächtnisambulanz (z.B. des ZfP Südwürttemberg in der Bethesda Klinik (Tel 0731 187 5317)).

Wir empfehlen darüber hinaus ergänzend zu den unter 1) genannten Therapiemaßnahmen, dass diese gezielt auch unter Doppel-Tätigkeit durchgeführt werden.

ad 7)

Auf Grund seines Alters, der Vorerkrankungen und der vorliegenden geriatrischen Syndrome besteht der V.a. eine Osteoporose Erkrankung.

Wir empfehlen zur weiteren Abklärung eine Vorstellung beim niedergelassenen Osteologen (z.B. Praxis Spiess, NU oder Praxis Radlmayer, Ulm) sowie die Durchführung einer Knochendichtemessung sowie ggf. die Einleitung einer spezifischen Osteoporosetherapie.

Eine Vitamin D Substitution wird bereits als Basistherapie durchgeführt.

Empfehlung an die weiterbehandelnden Ärzt*innen:

- Absetzen der Medikation mit Spasmex und Ibuprofen, Reduktion der Dosis von Metamizol auf 4x 500 mg

- Beginn einer Osteoporose-Prophylaxe mit Vitamin D (bspw. 1000 IE/tgl), Empfehlung zur Knochendichtemessung beim niedergelassenen Osteologen (z.B. Praxis Spiess, NU oder Praxis Radlmayer, Ulm), ggf. Beginn einer spezifischen Osteoporosetherapie

- wir bitten einen Antrag auf eine ambulante geriatrische Rehabilitation zu stellen.

- das Erstellen einer Vorsorgevollmacht und einer Patientenverfügung wurde der Tochter dringend empfohlen

Empfehlung an die weiterbehandelnden Therapeut*innen:

1. Die Durchführung einer ambulanten Rehabilitation mit umfangreichen Therapieangeboten der Physiotherapie, Ergotherapie und Bewegungstherapie. Die Antragsstellung läuft über Ihren Artz bzw. Ihre Ärtzin.

2. Die Teilnahme an einem gruppenbasiertes Sturzpräventionstraining mit den Schwerpunkten auf funktionellem Krafttraining und Gleichgewicht (3 x pro Woche).

Bei Fragen Kontaktaufnahme über 0731 187-181 (Telefon) oder sturzambulanz.bgu@agaplesion.de (Mail).

### Output

Sehr geehrter Herr Maier,

hier ist eine KI-generierte Übersetzung Ihres Arztbriefes aus der Sturzambulanz. Das Ziel ist es, Ihnen die wichtigsten Punkte in klaren, einfachen Worten zu erklären, damit Sie sich gut informiert und sicher fühlen.

**Zusammenfassung Ihrer wichtigsten Diagnosen:**

| **Diagnose** | **Erklärung** |
| --- | --- |
| **Multifaktorielle Gangstörung** | Ihre Schwierigkeiten beim Gehen haben mehrere verschiedene Ursachen, die zusammenwirken. |
| **Sensorisch-ataktische Gangstörung** | Durch Nervenschädigungen spüren Sie Ihren Untergrund nicht optimal, was das Gleichgewicht und die Koordination stört. |
| **Cerebrale Ischämie (mit Folgen)** | Ein früherer Schlaganfall im Gehirn führt dazu, dass Sie die Raumwahrnehmung und die Steuerung des linken Beins teilweise verloren haben. |
| **Leichte apraktische Gangstörung** | Ihr Gehirn hat zeitweise Schwierigkeiten, die komplexen Bewegungsabläufe beim Gehen zu planen, besonders wenn Sie gleichzeitig an etwas anderes denken. |

**Weitere bekannte Erkrankungen:**

- Polyneuropathie (Schädigung der peripheren Nerven)
- Netzhautablösung (schwere Sehbeeinträchtigung)
- Mild cognitive impairment (Verdacht auf leichte Gedächtniseinschränkungen)
- AV-Block III° (Herzrhythmusstörung, behandelbar durch Ihren Herzschrittmacher)
- Paroxysmales Vorhofflimmern (unregelmäßiger Herzschlag)
- Osteoporose (knochenschwund)
- Obstruktive Schlafapnoe (Atemaussetzer während des Schlafs)
- Arterielle Hypertonie (Bluthochdruck)
- Adipositas (starkes Übergewicht)
- Diabetes mellitus Typ 2 (Zuckerkrankheit)
- Presbyakusis (altersbedingte Schwerhörigkeit)

**Was wurde bisher gemacht?**

Sie wurden umfassend untersucht, um herauszufinden, warum Sie stürzen. Dabei zeigte sich, dass eine Kombination aus Nervenschäden, den Folgen eines Schlaganfalls, Sehproblemen und einer leichten Koordinationsstörung vorliegt. Sie haben bereits erfolgreich ein Training für Ausfall- und Abfangschritte (SturzImpfung) genutzt, was Ihre Sicherheit verbessert hat.

**Was sollten Sie jetzt tun?**

| **Maßnahme** | **Erklärung** | **Checkliste** |
| --- | --- | --- |
| **Ambulante Rehabilitation** | Beantragen Sie über Ihren Hausarzt eine Reha, um Physiotherapie, Ergotherapie und Bewegungstraining intensiv zu kombinieren. |  |
| **Sturzprävention** | Nehmen Sie an einem Gruppentraining (z. B. Ulmer Modell) teil oder nutzen Sie Alltagsübungen (LiFE), um Kraft und Gleichgewicht zu stärken. |  |
| **Ergotherapie** | Trainieren Sie in Einzelstunden Ihre Raumwahrnehmung, um im Alltag sicherer zu werden. |  |
| **Sehhilfe anpassen** | Nutzen Sie statt einer Gleitsichtbrille lieber zwei separate Brillen (eine für die Ferne, eine für die Nähe). |  |
| **Medizinische Abklärung** | Lassen Sie beim Neurologen weitere Tests (Blut und Nervenmessung) machen und kontrollieren Sie in 3–6 Monaten Ihre Gedächtnisleistung. |  |
| **Knochengesundheit** | Besuchen Sie einen Osteologen zur Knochendichtemessung, um die passende Therapie gegen Osteoporose zu finden. |  |
| **Medikamente prüfen** | Sprechen Sie mit Ihrem Arzt über das Absetzen von Spasmex und Ibuprofen sowie die Dosisanpassung von Metamizol. |  |
| **Hörgeräte** | Lassen Sie sich zur Versorgung mit Hörgeräten beraten. |  |

**Wichtige Kontaktdaten:**
Geriatrische Instituts- und Sturzambulanz:
📞 0731 187-181
📧 sturzambulanz.bgu@agaplesion.de
🌐 www.sturzimpfung-ulm.de
Gedächtnisambulanz ZfP Südwürttemberg: 📞 0731 187 5317

Ich hoffe, diese Übersetzung hilft Ihnen weiter. Bei Fragen oder Unsicherheit sprechen Sie bitte mit Ihrem Arzt.

Mit freundlichen Grüßen,

GISelA 🌼 (Ihre KI-Assistentin)

## English

### Input

Medical report for Mr. Maier:

Multifactorial gait disturbance
with 1) sensory-ataxic gait disturbance

due to 1A) Polyneuropathic syndrome of unclear genesis, DD nutritional DD idiopathic
Recommendation: further biochemical and electrophysiological clarification, therapy if applicable

1B) Secondary hemorrhagic cerebral ischemia, right parietal and temporal, 07/22
with residual left leg ataxia, suspected spatial perception disorder DD visual neglect
Recommendation: individual occupational therapy for spatial perception training and learning compensation strategies

1C) Status post retinal detachment OD (significant deficits) and OS (minor deficits)

2) suspected mild apractic gait disturbance

3) Suspected mild cognitive impairment
Abnormalities in dual-task gait; no abnormalities in cognitive screening
Recommendation: follow-up using the MoCA test in 3-6 months

4) Recurrent falls
most recently on June 16, 2024, with a fracture of the left os triquetrum.

5) Intermittent 3rd-degree AV block
Pacemaker implantation 06/2023

6) Paroxysmal atrial fibrillation
Anticoagulation with apixaban

7) Suspected osteoporosis

8) Severe obstructive sleep apnea

9) Arterial hypertension

10) Obesity

11) Type 2 diabetes mellitus, insulin-dependent

12) Suspected presbycusis
Hearing aid provision recommended

During the presentation at the Geriatric Institute and Fall Clinic, several components of a gait disturbance were observed:

ad 1A)
A sensory-ataxic gait disturbance was noted, which appears to be multifactorial in origin. A polyneuropathic syndrome of currently unclear genesis was observed.
For further clarification, we recommend biochemical diagnostics, particularly regarding potentially modifiable causes: Vitamin B12, holotranscobalamin, methylmalonic acid, Vit. B6, Vit B1, folic acid, serum electrophoresis (MGUS?), vasculitis screening (ESR, CRP; ANA, ANCA, Hepatitis C). An HbA1c value from a lab sample in 10/23 was slightly elevated at 6.4%; the patient reports weight gain and increased food intake since the cessation of regular alcohol consumption; therefore, more precise testing for diabetes should be considered.
We recommend an electrophysiological examination by a community neurologist to confirm the clinical diagnosis.
For the treatment of the sensory-ataxic gait component, we recommend: a structured fall prevention program, e.g., group-based according to the Ulm model (2-3x/week, 30-60 min) with targeted balance and strength exercises, with regular adjustment of the difficulty level to the individual level. Alternatively, the patient can integrate self-training into his daily routine, e.g., using LiFE training (www.life-alltagsuebungen.de).
Additionally, perturbation training, e.g., on a treadmill to train stepping and recovery strategies, is useful. This can be performed as part of the so-called "Fall Vaccination" (SturzImpfung) in our clinic and has already been used by the patient twice with subjective improvement in gait stability. For interest and questions, please contact our Fall Clinic via tel. 0731 187 181 or homepage www.sturzimpfung-ulm.de.

ad 1B) and 1C)
Clinical deficits in spatial perception and left leg ataxia were observed. These are attributable, on the one hand, to the prior high-parietal cerebral ischemia and, on the other, to the visual impairment including finger-perimetric visual field defects.
In addition to the therapeutic measures mentioned under ad 1), we recommend supplementary individual occupational therapy with the aim of improving spatial perception and, if applicable, learning compensation strategies.
Regarding the visual impairments, we recommend avoiding the use of varifocal glasses, which can also complicate the transition between near and far vision, and instead using separate glasses for distance and near vision.

ad 2)
A slight apractic gait component was noted, with deterioration of gait analysis parameters during semantic dual-task activities; however, this remained below the 20% cutoff. This may be due to subclinical cognitive deficits. Subjectively, the patient reports slowly progressive cognitive impairment, which is partially relevant to his daily life. A cognitive screening showed no measurable evidence of cognitive deficits.
For further clarification, we recommend a follow-up with a short cognitive test suitable for the patient's high initial cognitive level (e.g., standard MoCA test) in 3-6 months, as well as further observation if symptoms persist or progress, with a possible referral to a memory clinic (e.g., ZfP Südwürttemberg at the Bethesda Clinic, tel. 0731 187 5317).
Furthermore, we recommend that the therapies mentioned under 1) be specifically carried out under dual-task conditions.

ad 7)
Due to his age, previous illnesses, and existing geriatric syndromes, osteoporosis is suspected.
For further clarification, we recommend a presentation to a community osteologist (e.g., Praxis Spiess, NU or Praxis Radlmayer, Ulm), as well as a bone density measurement and, if applicable, the initiation of specific osteoporosis therapy.
Vitamin D supplementation is already being carried out as basic therapy.

Recommendations for the treating physicians:

- Discontinue medication with Spasmex and Ibuprofen; reduce the dose of Metamizole to 4x 500 mg
- Start osteoporosis prophylaxis with Vitamin D (e.g., 1000 IU/day); recommendation for bone density measurement by a community osteologist (e.g., Praxis Spiess, NU or Praxis Radlmayer, Ulm); initiate specific osteoporosis therapy if applicable
- We request that an application for outpatient geriatric rehabilitation be submitted.
- The daughter was strongly recommended to create a healthcare proxy (Vorsorgevollmacht) and a living will (Patientenverfügung).

Recommendations for the treating therapists:

1. Implementation of outpatient rehabilitation with extensive therapy offerings in physiotherapy, occupational therapy, and movement therapy. The application process is handled via the treating physician.
2. Participation in a group-based fall prevention training focusing on functional strength training and balance (3x per week).

For questions, please contact 0731 187-181 (phone) or sturzambulanz.bgu@agaplesion.de (email).

### Output

Dear Mr. Maier,

Here is an AI-generated translation of your medical report from the Fall Clinic. The goal is to explain the most important points to you in clear, simple words, so that you feel well-informed and secure.

Summary of your most important diagnoses:

| **Diagnosis** | **Explanation** |
| --- | --- |
| **Multifactorial gait disorder** | Your difficulties with walking have several different causes that interact with each other. |
| **Sensory-ataxic gait disorder** | Due to nerve damage, you cannot feel the ground beneath you optimally, which disturbs your balance and coordination. |
| **Cerebral ischemia (with sequelae)** | A previous stroke in the brain has led to a partial loss of spatial perception and control of the left leg. |
| **Mild apraxic gait disorder** | Your brain occasionally has difficulty planning the complex movement sequences involved in walking, especially when you are thinking about something else at the same time. |

**Other known conditions:**

- Polyneuropathy (damage to the peripheral nerves)
- Retinal detachment (severe visual impairment)
- Mild cognitive impairment (suspected mild memory impairment)
- AV block III° (heart rhythm disorder, treatable by your pacemaker)
- Paroxysmal atrial fibrillation (irregular heartbeat)
- Osteoporosis (bone loss)
- Obstructive sleep apnea (pauses in breathing during sleep)
- Arterial hypertension (high blood pressure)
- Obesity (severe overweight)
- Diabetes mellitus Type 2 (diabetes)
- Presbycusis (age-related hearing loss)

**What has been done so far?**

You have undergone a comprehensive examination to determine why you are falling. This showed a combination of nerve damage, the consequences of a stroke, visual problems, and a mild coordination disorder. You have already successfully participated in training for stepping and recovery strategies ("SturzImpfung"), which has improved your safety.

**What should you do now?**

| **Measure** | **Explanation** | **Checklist** |
| --- | --- | --- |
| Outpatient rehabilitation | Apply for rehabilitation via your primary care physician to intensively combine physiotherapy, occupational therapy, and exercise training. |  |
| Fall prevention | Participate in group training (e.g., Ulm Model) or use everyday exercises (LiFE) to strengthen strength and balance. |  |
| Occupational therapy | Train your spatial perception in individual sessions to become more confident in everyday life. |  |
| Adjust visual aids | Instead of progressive lenses, prefer using two separate pairs of glasses (one for distance, one for near vision). |  |
| Medical clarification | Have further tests (blood and nerve conduction measurements) performed by a neurologist and check your memory performance in 3–6 months. |  |
| Bone health | Visit an osteologist for a bone density measurement to find the appropriate therapy for osteoporosis. |  |
| Medication review | Speak with your doctor about discontinuing Spasmex and Ibuprofen, as well as adjusting the dose of Metamizole. |  |
| Hearing aids | Seek counseling regarding the provision of hearing aids. |  |

Important contact details:
Geriatric Institute and Fall Clinic:
📞 0731 187-181
📧 sturzambulanz.bgu@agaplesion.de
🌐 www.sturzimpfung-ulm.de
Memory Clinic ZfP Südwürttemberg: 📞 0731 187 5317

I hope this translation is helpful to you. If you have any questions or uncertainties, please speak with your doctor.

Sincerely,

GISelA 🌼 (Your AI Assistant)

# Few-Shot Prompt

**Note:** For data privacy and ethical reasons, the following few-shot prompt does not include concrete examples. In the actual application, these are implemented at the corresponding placeholders.

”””

<examples>

<example1>

<input>

[original letter]

</input>

<output>

[patient-friendly simplification]

</output>

</example1>

<example2>

<input>

[original letter]

</input>

<output>

[patient-friendly simplification]

</output>

</example2>

<example3>

<input>

[original letter]

</input>

<output>

[patient-friendly simplification]

</output>

</example3>

<example4>

<input>

[original letter]

</input>

<output>

[patient-friendly simplification]

</output>

</example4>

</examples>

###Aufgabe: Du bist GISelA, eine Übersetzerin von Arztbriefen in allgemeinverständliche Sprache. Der Nutzer legt Dir einen Arztbrief vor und Deine Aufgabe ist es, die Inhalte dieses Arztbriefes laiengerecht und allgemeinverständlich für eine betagte Person zu erklären. Benenne dazu die 3-5 wichtigsten Diagnosen und erkläre sie in einfachen Worten in Tabellenform, nimm dabei aber keine Bewertung vor. Fasse die restlichen Diagnosen in Stichpunkten zusammen und erkläre sie in einfachen Worten in einer Klammer dahinter. Beachte, dass die gesamte Zusammenfassung nicht länger als 400 Wörter sein sollte und an den Patienten adressiert ist. Sei in der Kommunikation einfühlsam. Rede nur über medizinische Inhalte und vermeide oder erkläre Fremdwörter und Abkürzungen.

Die gesamte Antwort ist medizinisch korrekt, aber ohne zu belehren.

Orientiere dich an folgendem Aufbau, zu erstellende Inhalte sind mit eckigen Klammern [] gekennzeichnet:

Sehr geehrte/r Frau/Herr [Name des Patienten],

hier ist eine KI-generierte Übersetzung Ihres Arztbriefes aus der Sturzambulanz. Das Ziel ist es, Ihnen die wichtigsten Punkte in klaren, einfachen Worten zu erklären, damit Sie sich gut informiert und sicher fühlen.

**Zusammenfassung Ihrer wichtigsten Diagnosen:**

[Tabelle mit Spalten Diagnose und Erklärung

Wichtig: Keine Interpretation oder Bewertung der Diagnosen!]

**Weitere bekannte Erkrankungen:**

[ein Absatz mit einer stichpunktartigen Auflistung der restlichen Diagnosen mit kurzer Erklärung in Klammern dahinter.

Beispiel: orthostatische Hypotension (Blutdruckabfall beim Aufstehen).]

**Was wurde bisher gemacht?**

[Zusammenfassung der Epikrise und Behandlung]

**Was sollten Sie jetzt tun?**

[Tabelle mit Auflistung der Empfehlungen und Erklärung, was das für den Patienten bedeutet, sowie einer dritten, leeren Spalte mit der Überschrift "Checkliste".

Die Empfehlungen müssen klar, handlungsorientiert und motivierend formuliert sein]

**Wichtige Kontaktdaten:**

Geriatrische Instituts- und Sturzambulanz:

📞 0731 187-181

📧 sturzambulanz.bgu@agaplesion.de

🌐 www.sturzimpfung-ulm.de

[wenn vorhanden: eventuelle E-Mail-Adressen und Telefonnummern aus dem Arztbrief]

Ich hoffe, diese Übersetzung hilft Ihnen weiter. Bei Fragen oder Unsicherheit sprechen Sie bitte mit Ihrem Arzt.

Mit freundlichen Grüßen,

GISelA 🌼 (Ihre KI-Assistentin)

“““

# Inter-rater Reliability

Inter-rater reliability between the two expert raters was assessed using the intraclass correlation coefficient (ICC, two-way random effects model, absolute agreement). The analysis was conducted on the item-level scores across all observations to evaluate the degree of consistency between raters in their application of the scoring instrument. The resulting ICC was −0.34, indicating poor agreement between experts, suggesting substantial variability in scoring behavior and limited interchangeability of ratings across raters. This finding implies that while the raters may show some degree of association in their scoring trends, their absolute agreement is insufficient for treating their scores as equivalent measurements. However, inferential comparisons in the main analysis were based on paired statistical tests, which do not require inter-rater interchangeability.

# Per-simplifier analyses

## Semantic Similarity

To validate the equivalence margin used in the primary analysis, the semantic fidelity of the AI-generated simplifications was compared against each expert simplifier individually using cosine similarity of sentence embeddings as a proxy. A paired *t*-test revealed no significant difference in semantic fidelity between the AI and Simplifier 1 (TDK; *d*=.070, 95% CI [-.313, .428], *P*=.694). While a significant difference was observed between the AI and Simplifier 2 (FMV; *d*=.748, 95% CI [.525, 1.074], *P*<.001), an independent t-test between the two expert simplifiers themselves also showed a significant difference in their semantic fidelity (*d*=.778, 95% CI [.266, 1.385], *P*=.003). These results indicate that the semantic variance between the AI and the human experts falls within the natural range of variability observed between expert simplifiers, thereby supporting the statistical equivalence established in the primary analysis.

# Figures and Table

## Figures

**Supplementary Figure S1: Overall quality scores for human authored simplifications.** Individual Likert-scaled items were aggregated to an overall score (range: 4-20 points). The patient representative mean score was 14.95 for simplifications authored by simplifier 1 and 17.27 for those authored by simplifier 2. Mean scores from the first expert rater were 16.77 (simplifier 1) and 17.89 (simplifier 2). Mean scores from the second expert rater were 14.52 (simplifier 1) and 13.92 (simplifier 2). Outliers were predominantly observed in patient representative scores for simplification authored by human simplifier 2. a. u. = arbitrary units

## Tables

**Supplementary Table S2: Simplifier 1 (TDK) main results.** The possible score range was 4–20 points. Mean scores and standard deviations (SD) are provided as evaluated by the patient group and two independent expert raters, including the corresponding *P* values for statistical significance, as well as Cohen's *d* and its 95% confidence interval.

| Rater | Mean ± SD (human) | Mean ± SD (AI) | Cohen's *d* | 95% CI | *P* value |
| --- | --- | --- | --- | --- | --- |
| Patient Representatives | 16.094 ± 3.888 | 17.688 ± 2.533 | -.409 | [-.701, -.089] | .027 |
| Expert 1 Sport Scientist | 16.625 ± 2.152 | 18.188 ± 1.306 | -.592 | [-.916, -.309] | .002 |
| Expert 2 Geriatrician | 14.281 ± 3.008 | 13.875 ± 2.744 | .104 | [-.247, .494] | .559 |

**Supplementary Table S3: Simplifier 2 (FMV) main results.** The possible score range was 4–20 points. Mean scores and standard deviations (SD) are provided as evaluated by the patient group and two independent expert raters, including the corresponding *P* values for statistical significance, as well as Cohen's *d* and its 95% confidence interval.

| Rater | Mean ± SD (human) | Mean ± SD (AI) | Cohen's *d* | 95% CI | *P* value |
| --- | --- | --- | --- | --- | --- |
| Patient Representatives | 13.812 ± 4.395 | 16.844 ± 3.611 | -.542 | [-.967, -.169] | .004 |
| Expert 1 Sport Scientist | 16.906 ± 1.692 | 17.594 ± 1.478 | -.295 | [-.697, .029] | .106 |
| Expert 2 Geriatrician | 14.750 ± 1.814 | 13.969 ± 1.892 | .325 | [-.015, .728] | .076 |

**Supplementary Table S4: Between simplifiers main results.** The possible score range was 4–20 points. Mean scores and standard deviations (SD) are provided as evaluated by the patient group and two independent expert raters, including the corresponding *P* values for statistical significance, as well as Cohen's *d* and its 95% confidence interval.

| Rater | Mean ± SD (human1) | Mean ± SD (human2) | Cohen's *d* | 95% CI | *P* value |
| --- | --- | --- | --- | --- | --- |
| Patient Representatives | 16.094 ± 3.888 | 13.812 ± 4.395 | .550 | [.078, 1.088] | .032 |
| Expert 1 Sport Scientist | 16.625 ± 2.152 | 16.906 ± 1.692 | -.145 | [-.620, .366] | .563 |
| Expert 2 Geriatrician | 14.281 ± 3.008 | 14.750 ± 1.814 | -.189 | [-.668, .346] | .453 |

**Supplementary Table S5: Simplifier 1 (TDK) individual items.** Median scores and interquartile ranges (IQR) are provided for specific assessment criteria, including clarity, appropriateness, positivity, completeness, and correctness, comparing human-authored and AI-generated simplifications as evaluated by the patient group and two expert raters, with accompanying *P* values for statistical significance, as well as the rank-biserial correlation coefficient (*r*) and its corresponding confidence interval.

| Rater | Item | Median (IQR) human | Median (IQR) AI | Rank-biserial *r* | 95% CI | *P* value |
| --- | --- | --- | --- | --- | --- | --- |
| Patient  Representatives | Clarity | 5 (3.75, 5.0) | 5 (4.0, 5.0) | -.604 | [-.927, -.033] | .048 |
|  | Appropriateness of the content | 4 (3.0, 5.0) | 4 (4.0, 5.0) | -.727 | [-1.000, -.181] | .030 |
|  | Positivity of the content | 4 (3.0, 5.0) | 5 (4.0, 5.0) | -.463 | [-.870, .091] | .096 |
|  | Appropriateness of the language | 4 (3.0, 5.0) | 5 (4.0, 5.0) | -.426 | [-.831, .134] | .123 |
| Expert 1 Sport Scientist | Clarity | 5 (4.0, 5.0) | 5 (5.0, 5.0) | -1.000 | [-1.000, -1.000] | .003 |
|  | Completeness | 4 (4.0, 5.0) | 4 (4.0, 5.0) | .190 | [-.300, .621] | .435 |
|  | Correctness | 4 (3.0, 4.0) | 4 (4.0, 5.0) | -.663 | [-.926, -.263] | .007 |
|  | Appropriateness of the language | 4 (3.75, 5.0) | 5 (4.0, 5.0) | -.805 | [-1.000, -.508] | .001 |
| Expert 2  Geriatrician | Clarity | 4 (3.75, 4.0) | 4 (3.0, 4.0) | -.290 | [-.572, .564] | .922 |
|  | Completness | 3 (3.0, 4.0) | 3 (2.0, 4.0) | .497 | [.105, .826] | .028 |
|  | Correctness | 3 (2.0, 4.0) | 3 (2.0, 4.0) | .133 | [-.333, .571] | .560 |
|  | Appropriateness of the language | 4 (3.0, 5.0) | 4 (4.0, 5.0) | -.542 | [-.897, -.013] | .055 |

**Supplementary Table S6: Simplifier 2 (FMV) individual items.** Median scores and interquartile ranges (IQR) are provided for specific assessment criteria, including clarity, appropriateness, positivity, completeness, and correctness, comparing human-authored and AI-generated simplifications as evaluated by the patient group and two expert raters, with accompanying *P* values for statistical significance, as well as the rank-biserial correlation coefficient (*r*) and its corresponding confidence interval.

| Rater | Item | Median (IQR) human | Median (IQR) AI | Rank-biserial *r* | 95% CI | *P* value |
| --- | --- | --- | --- | --- | --- | --- |
| Patient  Representatives | Clarity | 3 (3.0, 5.0) | 5 (4.0, 5.0) | -.637 | [-1.000, -.178] | .013 |
|  | Appropriateness of the content | 3 (3.0, 4.25) | 5 (3.0, 5.0) | -.621 | [-.909, -.216] | .010 |
|  | Positivity of the content | 3 (3.0, 5.0) | 5 (3.0, 5.0) | -.463 | [-.829, .027] | .060 |
|  | Appropriateness of the language | 3 (2.75, 4.0) | 4 (3.0, 5.0) | -.668 | [-.947, -.260] | .005 |
| Expert 1 Sport Scientist | Clarity | 4 (4.0, 4.0) | 5 (4.0, 5.0) | -.853 | [-1.000, -.600] | <.001 |
|  | Completeness | 5 (4.0, 5.0) | 4 (4.0, 4.0) | .917 | [.714, 1.000] | <.001 |
|  | Correctness | 4 (4.0, 5.0) | 4 (4.0, 5.0) | -.200 | [-.684, .228] | .394 |
|  | Appropriateness of the language | 4 (4.0, 4.0) | 5 (4.0, 5.0) | -.836 | [-1.000, -.532] | .001 |
| Expert 2  Geriatrician | Clarity | 3 (3.0, 4.0) | 4 (4.0, 4.0) | -.581 | [-.924, -.187] | .009 |
|  | Completness | 4 (3.0, 4.0) | 3 (2.0, 4.0) | .942 | [.779, 1.000] | <.001 |
|  | Correctness | 4 (3.75, 4.0) | 3 (3.0, 4.0) | .380 | [-.154, .908] | .141 |
|  | Appropriateness of the language | 4 (3.0, 4.0) | 4 (4.0, 4.0) | -.135 | [-.625, .396] | .591 |

**Supplementary Table S7: Between simplifiers individual items.** Median scores and interquartile ranges (IQR) are provided for specific assessment criteria, including clarity, appropriateness, positivity, completeness, and correctness, comparing human-authored and AI-generated simplifications as evaluated by the patient group and two expert raters, with accompanying *P* values for statistical significance, as well as the rank-biserial correlation coefficient (*r*) and its corresponding confidence interval.

| Rater | Item | Median (IQR) human1 | Median (IQR) human2 | Rank-biserial *r* | 95% CI | *P* value |
| --- | --- | --- | --- | --- | --- | --- |
| Patient  Representatives | Clarity | 5 (3.75, 5.0) | 3 (3.0, 5.0) | .315 | [.050, .568] | .021 |
|  | Appropriateness of the content | 4 (3.0, 5.0) | 3 (3.0, 4.25) | .213 | [-.068, .477] | .131 |
|  | Positivity of the content | 4 (3.0, 5.0) | 3 (3.0, 5.0) | .197 | [-.080, .454] | .162 |
|  | Appropriateness of the language | 4 (3.0, 5.0) | 3 (2.75, 4.0) | .328 | [.073, .583] | .020 |
| Expert 1 Sport Scientist | Clarity | 5 (4.0, 5.0) | 4 (4.0, 4.0) | .352 | [.107, .570] | .008 |
|  | Completeness | 4 (4.0, 5.0) | 5 (4.0, 5.0) | -.201 | [-.436, .030] | .112 |
|  | Correctness | 4 (3.0, 4.0) | 4 (4.0, 5.0) | -.251 | [-.478, -.024] | .051 |
|  | Appropriateness of the language | 4 (3.75, 5.0) | 4 (4.0, 4.0) | -.200 | [-.262, .268] | .994 |
| Expert 2  Geriatrician | Clarity | 4 (3.75, 4.0) | 3 (3.0, 4.0) | .265 | [.010, .523] | .046 |
|  | Completness | 3 (3.0, 4.0) | 4 (3.0, 4.0) | -.294 | [-.537, -.064] | .029 |
|  | Correctness | 3 (2.0, 4.0) | 4 (3.75, 4.0) | -.247 | [-.495, -.001] | .065 |
|  | Appropriateness of the language | 4 (3.0, 5.0) | 4 (3.0, 4.0) | .132 | [-.171, .398] | .328 |
